# Unilateral Papulopustular Dermatosis: Demodex Mites Bridging Rosacea and Demodicosis

**DOI:** 10.7759/cureus.79877

**Published:** 2025-03-01

**Authors:** Jesús Iván Martínez-Ortega, Tiffany Karoly Medina Angulo, Jacqueline E Mut Quej

**Affiliations:** 1 Dermatology, Dermatology Institute of Jalisco, Zapopan, MEX; 2 General Practice, Autonomous University of Campeche, Campeche, MEX; 3 Internal Medicine, Regional General Hospital No. 12 Lic. Benito Juárez, Yucatán, MEX

**Keywords:** atypical facial dermatoses, demodex folliculorum, demodex-related skin conditions, demodicosis, papulopustular demodicosis, papulopustular rosacea, skin immunology, topical ivermectin, unilateral facial dermatosis, unilateral rosacea

## Abstract

Papulopustular rosacea (PPR) is a common inflammatory dermatosis often associated with Demodex mite proliferation, though its pathogenesis remains incompletely understood. While bilateral presentations predominate, unilateral cases are rare and may offer unique insights into disease mechanisms. We report a case of a 68-year-old female with a chronic erythematous and scaly rash confined to the right malar region. Standardized skin surface biopsy revealed a high Demodex density (>5 mites/cm²), confirming demodicosis. Treatment with topical ivermectin cream resulted in significant improvement within four weeks. This case underscores the importance of considering demodicosis in the differential diagnosis of atypical facial dermatoses and revisits the classification, pathogenesis, diagnostic algorithm, and treatment strategies for inflammatory facial conditions. The findings support the inclusion of Demodex proliferation in disease frameworks and its potential impact on tailored therapeutic approaches.

## Introduction

Papulopustular rosacea (PPR) is a common inflammatory facial dermatosis characterized by vascular symptoms (flushing, persistent erythema, telangiectasia) and inflammatory features (papules and pustules). While its pathophysiology remains incompletely understood, evidence suggests a multifactorial process involving skin barrier compromise, dysregulated immune responses, heightened neurovascular reactivity, sebaceous gland hyperplasia, and eventual fibrosis [1-3].

*Demodex folliculorum* and *Demodex brevis*, commensal mites inhabiting pilosebaceous follicles, are frequently implicated in PPR. Although typically harmless, their abnormal proliferation or dermal invasion can cause demodicosis, raising questions about their role in PPR pathogenesis. High Demodex densities are observed in most PPR cases, and the condition often responds to acaricidal treatments. Evaluations based on modified Koch criteria, Hill criteria for causality, and the Rothman model further support the link between *Demodex *and PPR [2]. This connection has led to the hypothesis that demodicosis and PPR may represent a spectrum of the same disease [2]. Unilateral presentations, though rare, offer a unique opportunity to explore this hypothesis. Variations in microbial barriers-specifically Demodex distribution-may explain the localized involvement despite comparable skin barrier characteristics across facial compartments [4].

Here, we present a case of unilateral demodicosis in a 68-year-old woman with a chronic erythematous rash on the right malar region. A standardized skin surface biopsy demonstrated elevated Demodex densities, and the patient responded well to topical ivermectin cream. This case highlights the diagnostic and therapeutic challenges of unilateral facial dermatoses, emphasizing the importance of considering demodicosis in the differential diagnosis. A review of the existing literature further explores classification and connections with pathogenic, diagnostic, and treatment implications.

## Case presentation

A 68-year-old female patient presented with a chronic erythematous rash on the right malar region. She reported that the lesions had gradually worsened over the past six months, with intermittent episodes of exacerbation and partial remission. The patient noted increased pruritus and occasional burning sensations in the affected area. She mentioned occasional use of steroid-containing creams but denied any history of other dermatological conditions, use of other chemicals or creams, relevant family or significant medical history, or contact with pets or animals.

**Figure 1:**
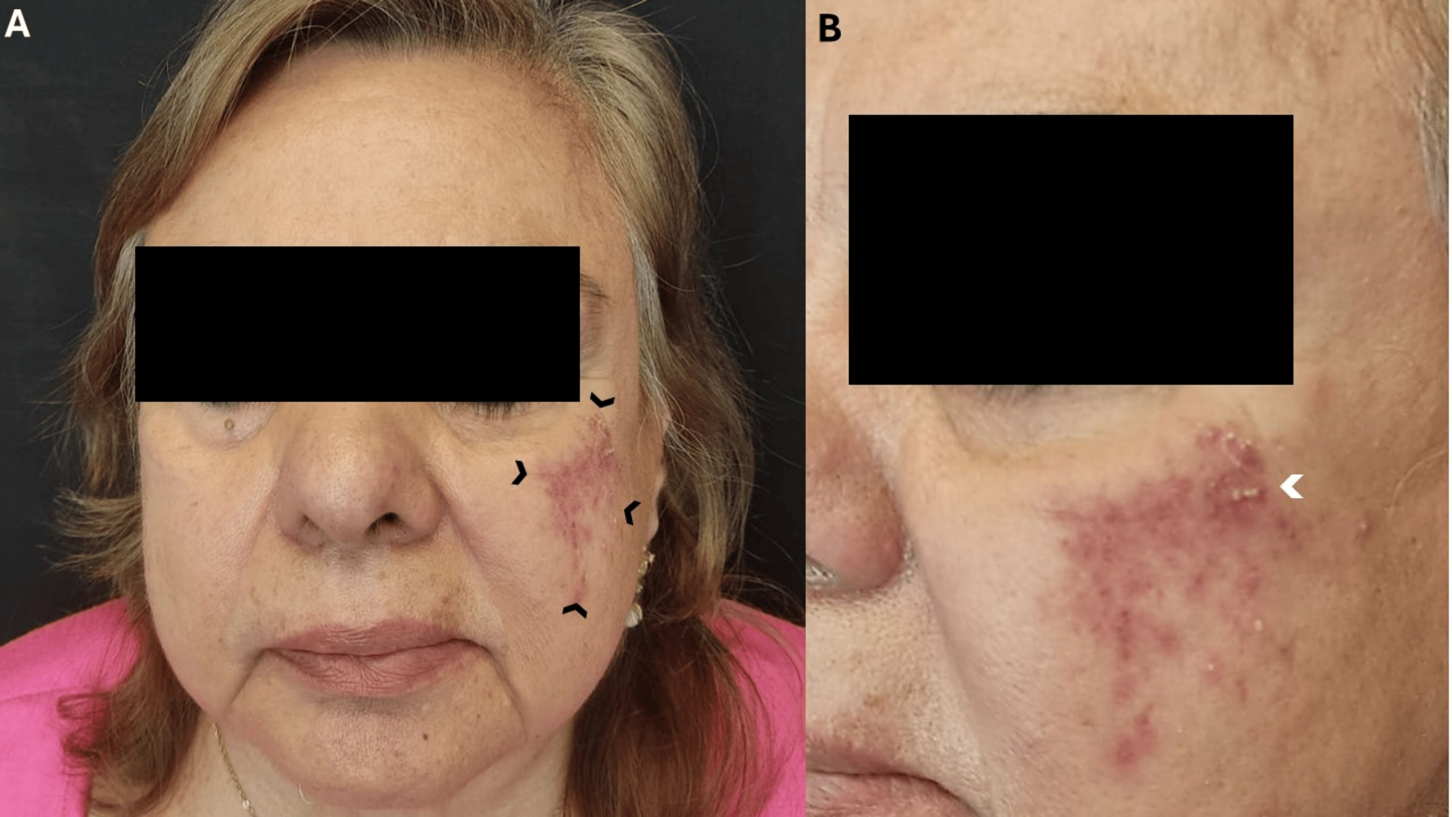
Clinical lesion. (A) Frontal view showing papules on an erythematous base localized to the right malar region (black arrows), consistent with a diagnosis of demodicosis. (B) Lateral view showing a closer view of the affected area, highlighting clusters of erythematous papules and pustules (white arrow) that correlate with increased density of Demodex mites, confirmed by microscopy.

On examination, multiple erythematous papules and pustules were observed on the right cheek, particularly in the malar area (Figures 1A, 1B). No other skin lesions were observed on the body.

Considering the clinical presentation of unilateral facial erythema and pustules, the primary differential diagnoses included demodicosis, bacterial folliculitis, contact dermatitis, and Malassezia folliculitis. A standardized skin surface biopsy was performed on the affected area, with microscopic examination revealing numerous *Demodex *spp. mites (Video 1), confirming the diagnosis of demodicosis. Laboratory tests were within normal limits. Routine laboratory tests, including a complete blood count (CBC) and blood chemistry panel, were performed, all within normal limits. The CBC showed normal leukocyte, erythrocyte, and platelet counts, while blood chemistry, including liver and renal function markers, was unremarkable.

**Video 1:** Standardized skin surface biopsy from the rash with microscopic examination showing at least six Demodex mites inside a follicular unit under light microscopy at 100x magnification in one field (threshold of ≥5 mites/cm² is consistent with demodicosis).

The patient was prescribed a topical ivermectin cream, which was applied twice daily. Follow-up at four weeks showed significant improvement in the lesions, with a decrease in erythema.

## Discussion

Rosacea is a chronic inflammatory skin disorder primarily affecting the central face, with a prevalence ranging from less than 1% to 22% globally [1,2]. Unilateral rosacea (UR), however, is rare and was first described by Shelley et al. in 1989 [5,6]. While data on UR are sparse, it may be underdiagnosed. A study of 244 patients identified 23 cases of unilateral involvement (excluding ocular rosacea) [7]. This raises important questions: “Does the anatomical distribution of rosacea influence diagnosis or treatment? Should cases dominated by Demodex mite proliferation be classified as demodicosis rather than rosacea?”

To better understand this unusual presentation, we refer to the literature review by Veraldi et al., who identified 11 cases using the terms “unilateral rosacea,” “unilateral demodicidosis,” and “unilateral Demodex sp. folliculitis” [5]. However, additional cases may exist under alternative terminology, such as “unilateral demodicosis,” “unilateral demodectic rosacea,” or “unilateral papulopustular rosacea.” Moreover, some cases may go unreported or may not be classified based on their anatomical distribution, but rather on other features, for instance, their morphology [8,9]. Others may be described using more specific anatomical terms, such as “periocular,” while still maintaining a unilateral presentation [9].

Demodicosis and rosacea: a spectrum of disease?

Demodicosis highlights a defined causative factor-Demodex mite overgrowth-while rosacea is diagnosed based on clinical features such as persistent centrofacial erythema, flushing, telangiectasia, and papules/pustules. Recent consensus guidelines have shifted from subtype-based classifications to phenotype-based approaches [2,3,10]. If a strong association between Demodex and PPR is confirmed, it could imply that PPR and demodicosis represent a spectrum of the same disease, with implications for diagnosis and treatment.

For instance, acaricidal treatments logically target Demodex-driven demodicosis, while the multifactorial nature of PPR may require broader strategies. Forton proposed integrating erythematotelangiectatic rosacea (ETR) and PPR into demodicosis phenotypes based on mite involvement [2]. Similarly, Chen and Plewig [11] categorized demodicosis into primary (immunocompetent) and secondary (immunosuppressed) forms, emphasizing clinical, immunological, and anatomical factors. Their classification includes anatomical distinctions, such as ocular demodicosis and auricular demodicosis [11]. Applying these frameworks, the current case would be classified as secondary papulopustular demodicosis.

Anatomical and clinical considerations

The inclusion of anatomical features in Chen and Plewig’s system highlights the potential value of further anatomical subclassifications. While their work focuses on specific anatomical regions (e.g., eyes and ears), extending this approach to consider unilateral versus bilateral facial involvement might offer additional insights [11]. For instance, localized presentations might indicate a more pronounced role for microbial or environmental triggers on the affected side, potentially necessitating tailored treatment strategies. Moreover, unilateral involvement might correlate with less severe systemic immune dysregulation, suggesting that such cases could respond well to more localized therapies [5].

Unilateral presentation: microbial and barrier considerations

In contrast to relatively uniform immunological and physical skin barriers, microbial and chemical barriers exhibit significant variability across anatomical regions [4,12]. For example, the cheeks harbor the highest density of Demodex mites, with notable differences even between symmetrical sites. Such variability may underlie the unilateral presentation of conditions like rosacea and demodicosis. External factors, such as prior corticosteroid use, can further disrupt the microbial barrier asymmetrically, promoting localized mite proliferation and inflammation [13]. These observations underscore the need for further research into how anatomical variations influence disease pathogenesis and treatment responses.

Recent findings have also deepened our understanding of the immunological barriers that maintain cutaneous homeostasis under normal conditions. Since Demodex mites are typically commensal, exploring the mechanisms that prevent their overgrowth is crucial for understanding their pathogenic roles. Notably, group 2 innate lymphoid cells (ILC2s) and interleukin-13 (IL-13) have been identified as key regulators of skin integrity. These components form an immune checkpoint that limits excessive Demodex colonization in hair follicles, as demonstrated in murine models [14].

Pathogenesis: immune and neurogenic interactions

Rosacea is primarily driven by Th1/Th17 immune responses, with significant contributions from macrophages, T-cells, fibroblasts, and mast cells (MCs). Activation of Toll-like receptor 2 (TLR2) by Demodex mites and their associated bacteria initiates an inflammatory cascade involving kallikrein-5 (KLK5) and cathelicidin LL-37, a peptide that further amplifies inflammation. LL-37 promotes MC degranulation through the Mrgprx2 receptor, leading to the recruitment of neutrophils, which release additional LL-37 and perpetuate the inflammatory cycle [1,3,15].

Fibroblasts also play a role by secreting cytokines such as CCL19 and CXCL1, which recruit CCR7+ T cells. Furthermore, fibroblasts upregulate prostaglandin D2 synthase (PTGDS), producing PGD2, a vasodilator associated with abnormal flushing [3], while their secretion of hyaluronic acid helps limit MC activation [16].

**Figure 2:**
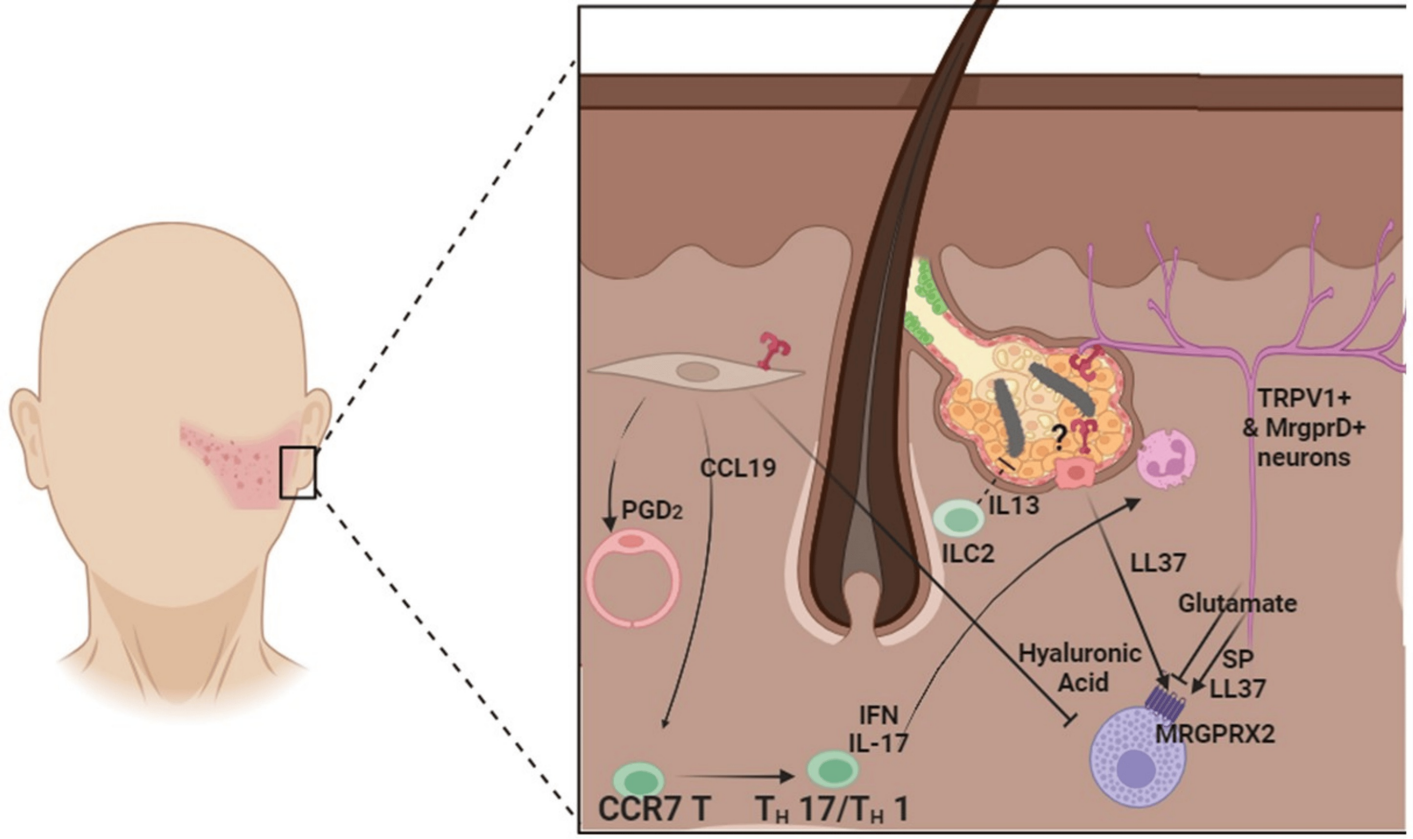
Immunological and cellular interactions in rosacea pathophysiology. This figure illustrates key molecular and cellular mechanisms involved in rosacea inflammation. Group 2 innate lymphoid cells (ILC2s, in green) and interleukin-13 (IL-13) play a critical role in maintaining skin integrity under normal conditions. However, a trigger or yet unidentified initiating factor can lead to Toll-like receptor 2 (TLR2, in red) activation. Several candidate ligands have been proposed, including Demodex mites, their excrement, and bacteria carried by the mites. TLR2 activation initiates KLK5 activation, which cleaves cathelicidin into LL-37 (in purple), a potent chemoattractant for mast cells (MCs). LL-37 induces MC degranulation. Mrgprx2 receptors on MCs further amplify this process by recruiting neutrophils, which release additional LL-37, perpetuating the inflammatory cycle.  Fibroblasts (in gray) play a central role in rosacea pathology by secreting cytokines such as CCL19, CXCL1, CXCL2, and CXCL12, which recruit CCR7+ T cells into lesional skin. Additionally, fibroblasts upregulate PTGDS, an enzyme that converts PGH2 into PGD2, a vasodilator contributing to the abnormal vasodilation characteristic of rosacea. This effect is mediated through the PGD2-PTGDR axis acting on endothelial cells (in pink). Fibroblast-derived hyaluronic acid also influences immune responses by regulating the A20/TNFAIP3 pathway, which limits MC activation.  Neurogenic factors further modulate inflammation: TRPV1-expressing neurons release substance P (SP), which, along with LL-37 produced by MrgprD-expressing neurons, activates MCs. In contrast, glutamate released by MrgprD-expressing neurons inhibits MC activation. Abbreviations: ILC2, group 2 innate lymphoid cells; IL-13, interleukin-13; TLR2, toll-like receptor 2; KLK5, kallikrein-5; LL-37, cathelicidin antimicrobial peptide; MC, mast cell; PTGDS, prostaglandin D2 synthase; PGD2, prostaglandin D2; PGH2, prostaglandin H2; CCR7, C-C chemokine receptor 7; SP, substance P. Image credits: Jesús Iván Martínez-Ortega.

Neurogenic factors further modulate rosacea pathogenesis. Substance P (SP) released by TRPV1-expressing neurons and LL-37 from MrgprD-expressing neurons can activate mast cells, while glutamate from these same neurons inhibits MC activation, underscoring the complex neuroimmune crosstalk involved in rosacea (Figure 2) [1,15].

These findings collectively emphasize the central role of mast cells in bridging immune and neurogenic pathways in rosacea pathophysiology. To maintain clarity and avoid oversaturation, other key immune actors, such as Langerhans cells and macrophages, as well as additional signaling molecules like VEGF - which contributes to the characteristic vasoactivity of rosacea - were not included in this figure. Nonetheless, these elements remain essential contributors to the complex inflammatory network underlying rosacea.

Diagnostic and therapeutic implications

**Figure 3:**
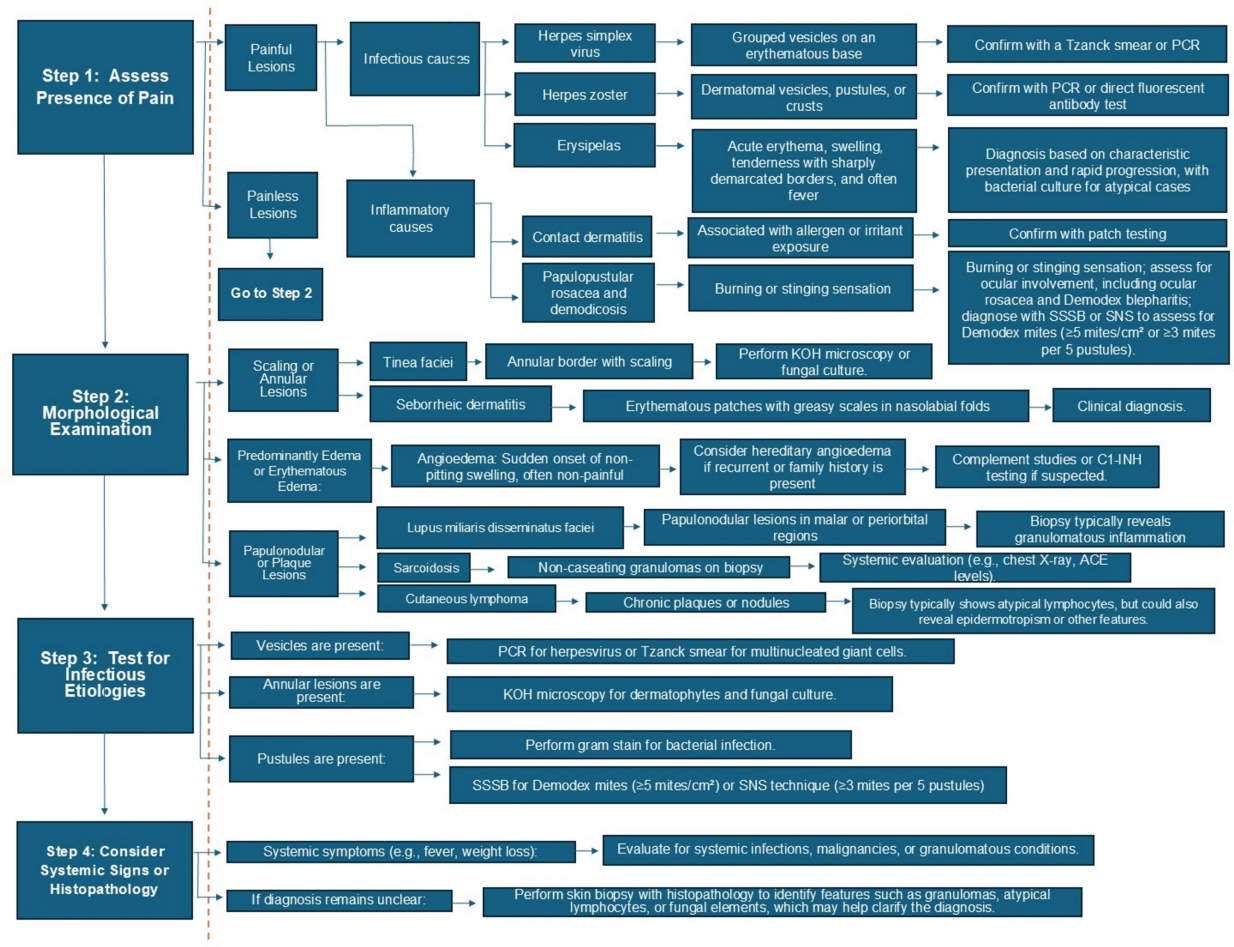
Algorithm for diagnosing unilateral erythematous facial lesions. A pivotal symptom, pain, is assessed in Step 1, as it is crucial in the initial diagnostic process. Painful lesions may suggest infectious or inflammatory causes such as herpes simplex virus, herpes zoster, erysipelas, contact dermatitis, papulopustular rosacea, or demodicosis. Notably, angioedema is characterized by rapid swelling without pain. Step 2 emphasizes the morphological examination of painless lesions, guiding the clinician through specific patterns such as scaling, annular, papulonodular, or plaque lesions. Steps 3 and 4 aim to confirm diagnoses or clarify unclear cases through targeted microbiological tests and histopathological analysis when systemic signs or diagnostic uncertainty persist. Abbreviations: PCR: Polymerase Chain Reaction, SSSB: Standardized Skin Surface Biopsy, ACE: Angiotensin-Converting Enzyme, C1-INH: C1 Esterase Inhibitor.

There is limited guidance available for the clinical differential diagnosis of unilateral erythematous facial lesions. To address this gap, we developed a diagnostic algorithm to assist clinicians in systematically evaluating and managing such presentations [17-19]. Less common unilateral occurrences of erythematous facial dermatoses, including lupus cutaneous, seborrheic pemphigus, and others, were excluded from the algorithm due to their rarity [20]. Similarly, systemic conditions such as climacteric flushing, drug reactions, cardiac disease, carcinoid syndrome, pheochromocytoma, mastocytosis, and anaphylaxis were omitted. Although malignancies like medullary thyroid carcinoma, pancreatic tumors, or renal carcinoma may occasionally present with facial erythema, systemic conditions involving the face generally manifest as bilateral erythema [17,18]. Additionally, it is worth noting that demodicosis can occasionally present without erythema or with subtle clinical features, as seen in pityriasis folliculorum [2]. Alternatively, it may manifest predominantly as pustules with minimal or no erythema. In such cases, the differential diagnosis should center on pustular dermatoses, including acne, bacterial folliculitis, acneiform eruptions, and Malassezia folliculitis (Figure 3) [21,22].

Treatment strategies for unilateral demodicosis are largely extrapolated from PPR guidelines. According to the 2017 Global Rosacea Consensus (ROSCO), recommended options include topical ivermectin, metronidazole, and azelaic acid, as well as oral doxycycline or isotretinoin [10]. For localized presentations, topical therapies may suffice, but further research is needed to validate this approach. Limitations of this study include the absence of follow-up imaging and its single-case nature, which may limit generalizability.

## Conclusions

This case of unilateral facial dermatosis, characterized by high Demodex density and a favorable response to acaricidal treatment, highlights the importance of considering localized demodicosis in the differential diagnosis of atypical inflammatory facial lesions. Substantial evidence supports the role of Demodex mites in the pathogenesis of PPR, aligning with established causality models. The unilateral presentation underscores the influence of localized microbial distribution and skin barrier variations in disease expression. While Demodex overgrowth appears central to disease expression in specific contexts, such as PPR or demodicosis, further studies are needed to elucidate its role as a primary factor, disease modifier, or secondary phenomenon in varying clinical scenarios. Refining these distinctions will be essential for improving diagnostic accuracy and optimizing treatment strategies, particularly in atypical presentations.
